# Traumatic brain injury and occupational risk of low-level blast exposure on adverse career outcomes: an examination of administrative and medical separations from Service (2005–2015)

**DOI:** 10.3389/fneur.2024.1389757

**Published:** 2024-04-16

**Authors:** Jennifer N. Belding, James Bonkowski, Robyn Englert

**Affiliations:** ^1^Naval Health Research Center, San Diego, CA, United States; ^2^Leidos, Inc., San Diego, CA, United States

**Keywords:** blast, low-level blast, traumatic brain injury, concussion, military, epidemiology, medical separation, job loss

## Abstract

**Introduction:**

Although traumatic brain injury (TBI) has been linked with adverse long-term health, less research has examined whether TBI is linked with non-clinical outcomes including involuntary job loss. Symptoms associated with TBI may influence one’s ability to maintain gainful employment including employment in the U.S. military. That influence may impact military service members with exposure to repetitive low-level blast (LLB). Understanding the association between TBI and involuntary job loss outcomes among military populations is particularly important as it may be associated with differences in eligibility for post-service benefits. The purpose of the present research was to determine whether (1) TBI and related conditions are associated with involuntary job loss (i.e., medical and administrative separations from service) among military personnel, and (2) occupational risk of LLB is associated with involuntary job loss in both the presence and absence of clinical diagnoses of TBI and related conditions.

**Method:**

This research leveraged population-level data from the Career History Archival Medical and Personnel System for enlisted personnel who served on active duty between 2005–2015. Risk of LLB exposure was categorized using military occupational specialty as a proxy. Medical diagnoses were identified using ICD-9 codes. Separations for medical and administrative reasons were identified.

**Results:**

Risk for administrative separation differed across medical diagnoses of interest, but those who worked in high-risk occupations were more likely to be administratively separated than those working in low-risk occupations. Risk for medical separation was associated with occupational risk of LLB and each of the diagnoses of interest, though significant interactions suggested that the effects of certain diagnoses of interest (e.g., concussion, cognitive problems, postconcussive syndrome, migraines) on medical separations was greater among those working in high-risk occupations.

**Discussion:**

Taken together, the present research suggests that TBI and associated medical conditions, as well as occupational risk of LLB, are associated with long-term involuntary job loss for medical reasons. This study is the first to demonstrate involuntary military job loss outcomes associated with TBI, mental health conditions, and conditions associated with blast exposure using both inpatient and outpatient population-level data and may have important implications for civilian employment and post-service benefits.

## 1 Introduction

The rapidly expanding field of research on traumatic brain injury (TBI) has documented the associations between TBI and long-term health and well-being (1–6). Although focus on mental and behavioral health outcomes associated with TBI is common, a relatively smaller body of research has examined the nature of the relationship between TBI and non-clinical outcomes including workplace outcomes (e.g., lost income potential, job loss) (3, 7–11). For example, previous research suggests that TBI severity is associated with reduced likelihood of returning to work post-injury (8, 12). Whereas nearly two thirds of patients with a loss of consciousness for an hour or less returned to work within one year, only a third of patients who were unconscious for one to two weeks were able to return to work within the same timeframe (12).

A variety of explanations have been offered for why patients experience such adverse workplace outcomes (12). For example, TBI and related conditions may be associated with cognitive impairments (e.g., delays in cognitive processing, memory lapses) as well as affective symptoms (e.g., irritability, impaired decision making) which can affect performance (9, 13, 14). In one study using qualitative interviews, patients with TBI self-reported that memory problems and fatigue were leading challenges that affected their ability to return to work following TBI (11). In another study investigating the effects of severe TBI on difficulty returning to work, patients reported more concerns with cognitive complaints than somatic complaints (e.g., balance, motricity, headaches) 8 years following injury (3). Furthermore, patients with concussion (often referred to as mild TBI [mTBI]), compared to those without concussion, may be less likely to be gainfully employed or work in management positions approximately five years later. This may be due in part to reduced capacity to handle one’s workload (9). However, many studies of workplace outcomes associated with TBI were limited to moderate to severe TBIs, were based on relatively small samples, or were unable to account for a variety of conditions that are often comorbid with TBI (e.g., posttraumatic stress disorder [PTSD]) (8). According to a literature review of long-term functional outcomes associated with TBI, findings specifically on the association between concussion and involuntary job loss have been termed “insufficient or contradictory” (p. 649) (8) and additional research is needed to understand the scope of potential workplace outcomes associated with TBI.

TBI is a prevalent condition among Service members in the U.S. Armed Forces. Estimates provided by the Traumatic Brain Injury Center of Excellence suggests more than 470,000 service members sustained a TBI between 2000 and 2022 (15). Additionally, estimated rates of sustained TBI among service members range between 11–20% (16–18). Due to this high prevalence of TBI among military populations, coupled with high performance requirements for military duties and missions (15, 19–21), understanding the employment outcomes associated with TBI among military personnel is of unique importance. For example, difficulty concentrating and delayed reaction time may have detrimental effects in a combat zone when under fire. Investigation of workplace outcomes is also unique in this population because service members who experience symptoms may seek comprehensive medical care at no cost. Due to Department of Defense (DoD) data management policies, comprehensive records of medical and career events (e.g., diagnoses, promotions) are maintained for all active duty service members throughout their time in service. This access to no cost medical care (thus removing a potential barrier to healthcare utilization), combined with complete archival records create an ideal study population for population-level epidemiological investigations of the association between TBI and employment outcomes, including involuntary job loss.

Involuntary military job loss (i.e., being involuntarily and prematurely separated from military service) for service members is often categorized into either medical separations (i.e., involuntary job loss due to inability to perform necessary job functions due to illness or injury) or administrative separations (i.e., termination for cause, such as illegal behavior) (22–24). Those separated for the former are typically eligible for post-service benefits (e.g., healthcare coverage, disability pay), whereas those separated for the latter are typically not (25, 26). While both types of involuntary job loss are associated with a variety of implications for the service member and the service branches (e.g., undermanning), medical separations may carry a unique burden to the taxpayer over time as those medically separated for service-related injuries may continue to receive medical care through the Veterans Health Administration (25, 27).

Relatively limited research to date has specifically examined associations between TBI and medical and administrative separations from military service (8). One previous study that leveraged hospitalization medical records from 1992 showed that concussion (vs. no concussion) was associated with significantly greater risk of administrative separations (e.g., due to behavioral discharges, criminal convictions, substance abuse) and significantly greater risk of medical discharge (28). Although this observation was important, this study may not be representative of those with concussion for several reasons. First, the majority of people with concussion do not seek medical care or are treated on an outpatient basis and are thus not often hospitalized, so the mTBIs in this cohort may have been more severe than the average mTBI (29–31). Second, because screening, diagnosis, and treatment of TBI in the military has dramatically changed over the past 25 years, it is likely that these previous findings are not representative of the modern association between TBI (including concussion as well as more severe TBIs) and involuntary military occupation loss (18, 19, 21). For example, the Department of Defense Instruction 6490.11, Defense Health Agency Procedural Instruction 6490.04, and the Military Acute Concussion Evaluation 2 were developed during this time period and have resulted in changes to screening, diagnosis, and treatment of TBI among military personnel (32).

Although TBIs among military and civilian populations have many similarities (e.g., causes, sequelae), there are several risk factors for TBI and involuntary job loss that are unique to military service. For example, previous research suggests that service members may be exposed to low-level blast (LLB; i.e., blast exposure which occurs from the firing of heavy weapons during both training and deployed environments (33)), which may increase their risk of sustaining a TBI and reporting corresponding symptoms (34–36). LLB exposure has been linked to a variety of symptoms (e.g., trouble hearing, headaches, memory loss) that may have important implications for employment (34, 37). Furthermore, military personnel may be more likely than their civilian counterparts to be diagnosed with other health issues that are associated with TBI (e.g., mental health conditions) and may play a role in their ability to maintain gainful employment (9, 38, 39). Due to the requirements of military service which often necessitate peak physical and mental performance, it is possible that the combination of one’s medical condition and occupation-based duty expectations may interact to influence involuntary military job loss. However, such hypotheses have not been examined to date.

The purpose of the present research was to determine whether (1) TBI and conditions commonly comorbid with TBI are associated with involuntary military job loss (i.e., medical and administrative separation from service) among military personnel, and (2) occupational risk of LLB is associated with involuntary military job loss in both the presence and absence of clinical diagnoses of TBI and related conditions. To answer these questions, the present research extended work previously reported by Belding and colleagues which documented associations between occupational risk of LLB and TBI and related conditions, particularly among those with greater time in service (40). In the present research, we examined whether clinical diagnoses of TBI and corresponding conditions and occupational risk of LLB were associated with medical and administrative separations.

## 2 Materials and methods

### 2.1 Data source and participants

The present research leveraged data from the Naval Health Research Center’s Career History Archival Medical and Personnel System (CHAMPS) database (41). This database maintains a longitudinal record of service members’ pay-affecting career records and medical diagnoses recorded during Tricare-reimbursable medical encounters in military- and civilian-based care settings. Service members were eligible for inclusion if they initially enlisted in the U.S. military between 1 October 2005 and 30 September 2014 and served for at least one year in the Army, Air Force, Marine Corps, or Navy. They were followed until one of the following occurred: they changed service branches, they became warrant officers or officers, their occupation changed that resulted in a risk categorization shift, they were discharged from military service for at least 30 days, or the study window end date of 31 December 2015. Eligible participants were classified as being at low, moderate, or high occupational risk of repetitive LLB exposure based on their military occupational specialty (categorized using Department of Defense-wide naming conventions) as described in Belding et al. (40). High-risk occupations included general armor and amphibious; artillery and gunnery; aviation ordnance; general combat engineering; general combat operations control; explosive ordnance disposal/Underwater Demolition Team; expeditionary medical services; general infantry; infantry, gun crews, seamanship specialists; military training instructor; missile artillery operating crew; rocket artillery; and special forces. Moderate-risk jobs included ammunition repair, artillery repair, counterintelligence, general armament maintenance, general law enforcement, independent duty hospital services, operational intelligence, security guards, and tracked vehicles. The remaining 160 occupations were categorized as low-risk.

### 2.2 Medical diagnoses

Diagnoses of interest included TBI, conditions commonly comorbid with concussion, behavioral health conditions, and blast-related conditions (see Table 1). TBI diagnoses were examined together, as well as separately by severity (i.e., mild, moderate, severe, penetrating), in accordance with case criteria established by the Armed Forces Health Surveillance Branch Division (42). Conditions commonly comorbid with concussion and behavioral health conditions were identified using case criteria from a recent RAND report on concussion in the military (19). Diagnoses commonly comorbid with concussion included alteration in mental status, cognitive problems, communication disorders, dizziness/vertigo, gait and coordination problems, headache, hearing problems, non-headache pain, skin sensation disturbances, sleep disorders/symptoms, smell and taste disturbances, syncope/collapse, and vision problems. Behavioral health conditions included adjustment disorders, anxiety disorders, acute stress disorders, alcohol abuse/dependence, attention-deficit/hyperactivity disorder (ADD/ADHD), bipolar disorder, delirium/dementia, depression, drug abuse/dependence, personality disorders, and PTSD. Blast-related conditions were identified consistent with Belding et al., and included postconcussive syndrome, tinnitus, fatigue, and migraines (40). For each medical diagnosis or category of interest, the date of earliest diagnosis on record and whether the encounter occurred in inpatient or outpatient settings were identified. Specific ICD-9 codes are available in the supplement to Belding et al. (40).

**Table 1:** Sample characteristics.

	N Total *N* = 1,336,845	%
Branch of service		
Air Force	226,847	17.0
Army	553,747	41.4
Marine Corps	261,379	19.6
Navy	294,872	22.1
Occupational Risk for LLB		
Low	906,635	67.8
Moderate	110,470	8.3
High	319,740	23.9
Sex		
Male	1,132,076	84.7
Female	204,769	15.3
Status at end of study window		
Administrative separation	172,927	13.0
Medical Separation	94,375	7.0
Early Release	56,260	4.2
End of active service	305,934	22.9
Not discharged	698,570	52.3
Other	7,255	0.5
Reenlistment	1,457	0.1
Retirement	67	<0.1
Number of SMs with Diagnosis		
TBI		
Any TBI	91,273	7.5
Mild TBI	91,273	6.8
Moderate TBI	18,499	1.4
Severe TBI	617	<0.1
Penetrating TBI	907	<0.1
Conditions comorbid with concussion		
Altered mental status	20,729	1.6
Cognitive problems	42,108	3.1
Communication problems	3,732	0.3
Dizziness/vertigo	8,767	0.7
Gait and coordination problems	812	0.1
Headache	122,656	9.2
Hearing problems	87,862	6.6
Nonheadache pain	792,865	59.3
Skin sensation disturbances	1,129	0.1
Sleep disorders and symptoms	248,275	18.6
Syncope and collapse	3,295	0.2
Vision problems	49,530	3.7
Mental health conditions		
Any behavioral health condition	380,961	28.5
Adjustment disorders	162,458	12.2
Anxiety disorders	154,619	11.6
Acute stress disorders	780	0.1
Alcohol abuse/dependence	142,700	10.7
ADD/ADHD	38,385	2.9
Bipolar disorder	13,351	1.0
Delirium/dementia	17,664	1.3
Depression	74,962	5.6
Drug abuse/dependence	51,161	3.8
Personality disorders	17,891	1.3
PTSD	60,342	4.5
Blast-related conditions		
Postconcussive syndrome	17,092	1.3
Tinnitus	49,514	3.7
Fatigue	77,788	5.8
Migraine	74,827	5.6

### 2.3 Involuntary military job loss

When service members separate from military service, the CHAMPS database retains the official reason for separation. A total of 137 reasons were recorded which were categorized into one of the following mutually exclusive groups: administrative separation (e.g., “court-martial,” “failure to meet minimum qualifications for retention”), medical separation (e.g., “disability, severance pay,” “death, cause not specified”), early release (e.g., “early release, in the national interest”), end of active service (e.g., “expiration of term of service”), other (e.g., “conscientious objector,” “record correction”), reenlistment (e.g., “immediate reenlistment”), and retirement (e.g., “retirement, 20 to 30 years of service”). Those who were not separated were coded as “not discharged.”

### 2.4 Statistical analyses

Descriptive statistics were used to characterize the sample. Whether service members were medically or administratively separated, respectively, was regressed on flags for each diagnosis of interest, occupational risk of LLB, and their interaction while adjusting for branch of service (referent: Air Force) and sex (referent: males) in separate Cox proportional hazard models. Due to the number of comparisons and large sample size, a strict threshold of *p* ≤ 0.001 was used to determine statistical significance for omnibus tests. When significant interactions emerged, we decomposed them by rerunning the Cox proportional hazard models stratifying by occupational risk and compared 95% confidence intervals for overlap. Non-overlapping confidence intervals were interpreted as being significantly different. We opted to utilize 95% confidence intervals (rather than alternatives) for ease of comparison of our findings with those reported in other papers. Although this means the significance threshold for omnibus effects differs from post-hoc analyses, we felt that this decision was justified by the fact that we had specific directional *a priori* hypotheses and different thresholds for significance in decomposing interactions is inherent in calculations that adjust for family-wise error rates.

## 3 Results

Characteristics of the final study sample are presented in Table 1. The sample was predominantly male and approximately 41% served in the Army, with the remainder relatively evenly split among the Air Force, Marine Corps, and Navy. The majority of service members worked in low-risk occupations; approximately 24 and 8% of the sample worked in high- and moderate-risk occupations, respectively. Nearly half of the sample was separated at the end of the study window. The average length of service by the end of the study window was 4.2 years (SD = 2.2 years).

### 3.1 Administrative separation

Across the board, occupational risk of LLB was associated with greater risk of being administratively separated from service (ORs ranged from 1.03–1.06; see Table 2). In contrast, medical diagnoses were associated with greater risk, no difference in risk, or less risk depending on the condition. For example, any TBI, mild TBI, and moderate TBI were all associated with significantly less risk of being administratively separated, whereas severe and penetrating TBI were not associated with differences in administrative separation at the *p* < 0.001 level. Among the conditions commonly comorbid with concussion, all were associated with decreased risk of being administratively separated, except for altered mental status and syncope and collapse, which were both associated with significantly greater risk. With the exception of delirium/dementia, all of the behavioral health conditions were associated with significantly greater risk of being administratively separated. One particularly notable finding is that those with (vs. without) drug abuse/dependence conditions were more than 5 times more likely to be administratively separated. A similar finding was noted with personality disorders such that those with (vs. without) the disorder were more than 4 times more likely to be administratively separated. Each of the blast-associated conditions were associated with significantly less likelihood of being administratively separated.

**Table 2:** Results from Cox regression analyses showing associations of occupational risk for LLB, diagnoses, and their interaction on administrative separations when adjusting for number of years in the study window, branch of service, and sex.

	Occupational LLB risk				Diagnosis				Interaction			
	OR	95% CI LL	95% CI UL	*p*	OR	95% CI LL	95% CI UL	*p*	OR	95% CI LL	95% CI UL	*p*
TBI												
Any TBI	1.06	1.05	1.06	*	0.91	0.90	0.93	*	0.89	0.88	0.91	*
Mild TBI	1.05	1.05	1.06	*	0.95	0.93	0.97	*	0.90	0.89	0.92	*
Moderate TBI	1.05	1.04	1.06	*	0.68	0.66	0.71	*	0.82	0.79	0.86	*
Severe TBI	1.04	1.04	1.05	*	0.67	0.52	0.86	0.002	0.92	0.70	1.19	0.51
Penetrating TBI	1.04	1.04	1.05	*	0.74	0.61	0.89	0.002	0.84	0.69	1.02	0.08
Conditions comorbid with concussion												
Altered mental status	1.04	1.04	1.05	*	1.69	1.64	1.74	*	0.97	0.94	1.00	0.06
Cognitive problems	1.05	1.05	1.06	*	0.58	0.56	0.59	*	0.92	0.89	0.95	*
Communication problems	1.04	1.04	1.05	*	0.52	0.47	0.58	*	0.80	0.72	0.89	*
Dizziness/vertigo	1.04	1.04	1.05	*	0.67	0.63	0.73	*	1.05	0.97	1.31	0.25
Gait and coordination problems	1.04	1.04	1.05	*	0.76	0.62	0.93	*	1.10	0.89	1.36	0.40
Headache	1.05	1.04	1.05	*	0.77	0.76	0.79	*	0.94	0.92	0.96	*
Hearing problems	1.06	1.06	1.07	*	0.59	0.57	0.60	*	0.91	0.89	0.93	*
Nonheadache pain	1.03	1.02	1.04	*	0.74	0.74	0.75	*	1.01	0.99	1.02	0.36
Skin sensation disturbances	1.04	1.04	1.05	*	0.70	0.58	0.84	*	0.84	0.69	1.02	0.08
Sleep disorders and symptoms	1.06	1.05	1.06	*	0.99	0.97	1.00	0.01	0.95	0.94	0.96	*
Syncope and collapse	1.04	1.04	1.05	*	1.43	1.32	1.55	*	1.13	1.04	1.23	0.005
Vision problems	1.04	1.03	1.05	*	0.74	0.71	0.76	*	1.04	1.01	1.08	0.009
Mental health conditions												
Any behavioral health condition	1.03	1.02	1.04	*	2.87	2.84	2.90	*	1.02	1.01	1.03	0.001
Adjustment disorders	1.06	1.05	1.06	*	2.03	2.01	2.06	*	0.97	0.96	0.99	*
Anxiety disorders	1.05	1.04	1.05	*	1.30	1.28	1.32	*	0.98	0.97	0.99	0.002
Acute stress disorders	1.04	1.04	1.05	*	2.18	1.91	2.49	*	0.99	0.86	1.13	0.85
Alcohol abuse/dependence	1.04	1.03	1.05	*	2.92	2.89	2.95	*	0.97	0.96	0.98	*
ADD/ADHD	1.05	1.04	1.05	*	1.39	1.36	1.43	*	1.00	0.97	1.02	0.73
Bipolar disorder	1.04	1.04	1.05	*	2.45	2.37	2.53	*	1.00	0.97	1.03	0.91
Delirium/dementia	1.05	1.04	1.05	*	0.77	0.74	0.80	*	0.85	0.81	0.88	*
Depression	1.05	1.04	1.05	*	2.21	2.17	2.24	*	1.00	0.99	1.02	0.93
Drug abuse/dependence	1.04	1.03	1.04	*	5.29	5.22	5.36	*	1.00	0.99	1.01	0.96
Personality disorders	1.05	1.04	1.05	*	4.51	4.40	4.62	*	1.02	1.00	1.05	0.06
PTSD	1.05	1.04	1.06	*	1.03	1.01	1.05	*	0.93	0.91	0.95	*
Blast-related conditions												
Postconcussive syndrome	1.05	1.04	1.05	*	0.76	0.73	0.79	*	0.84	0.81	0.88	*
Tinnitus	1.05	1.05	1.06	*	0.53	0.51	0.54	*	0.96	0.94	0.99	0.012
Fatigue	1.04	1.04	1.05	*	0.72	0.71	0.74	*	0.96	0.94	0.99	0.007
Migraine	1.04	1.04	1.05	*	0.81	0.79	0.83	*	0.96	0.93	0.98	0.001

*p < 0.001.

Of most importance, significant interactions emerged for several conditions including any TBI, mild TBI, moderate TBI, cognitive problems, communication problems, headache, hearing problems, any behavioral health problem, adjustment disorders, alcohol abuse/dependence, delirium/dementia, PTSD, postconcussive syndrome, and migraine (see Table 3). These significant interactions were decomposed by comparing 95% confidence intervals as a function of occupational risk. With only one exception (any behavioral health condition), the diagnosis of interest was associated with significantly less likelihood of being administratively separated from service among those in high-risk occupations relative to lower risk occupations. However, we note that planned comparisons were not always statistically significantly different at each level. Stated differently, while odds ratios were always lower among those in high-risk occupations than moderate or low-risk occupations, it was not always significantly lower than both. It is also worth noting that odds ratios for adjustment disorders and alcohol abuse/dependence were still greater than 1.0, whereas the others were all lower than 1.0.

**Table 3:** Decomposition of significant interactions of occupational risk of LLB and diagnoses of interest on administrative separations when adjusting for number of years in the study window, branch of service, and sex.

Effect of diagnosis among	Low LLB risk				Moderate LLB risk				High LLB risk			
	OR	95% CI LL	95% CI UL	*p*	OR	95% CI LL	95% CI UL	*p*	OR	95% CI LL	95% CI UL	*p*
TBI												
Any TBI	1.02	1.00	1.04	0.11	1.00	0.94	1.06	0.98	0.80	0.78	0.83	*
Mild TBI	1.04	1.02	1.07	*	1.03	0.97	1.10	0.31	0.85	0.82	0.87	*
Moderate TBI	0.83	0.79	0.88	*	0.68	0.57	0.79	*	0.56	0.53	0.60	*
Conditions comorbid with concussion												
Cognitive problems	0.62	0.60	0.65	*	0.65	0.59	0.73	*	0.52	0.50	0.55	*
Communication problems	0.65	0.57	0.75	*	0.43	0.27	0.66	*	0.42	0.35	0.51	*
Headache	0.82	0.80	0.84	*	0.87	0.82	0.92	*	0.71	0.69	0.74	*
Hearing problems	0.64	0.63	0.66	*	0.62	0.57	0.67	*	0.53	0.51	0.55	*
Sleep disorders and symptoms	1.03	1.02	1.05	*	1.08	1.04	1.12	*	0.92	0.90	0.94	*
Mental health conditions												
Any behavioral health condition	2.82	2.78	2.85	*	2.67	2.58	2.77	*	2.97	2.92	3.03	*
Adjustment disorders	2.08	2.05	2.11	*	2.12	2.04	2.21	*	1.96	1.92	2.00	*
Alcohol abuse/dependence	3.00	2.96	3.04	*	2.97	2.86	3.09	*	2.84	2.79	2.90	*
Delirium/dementia	0.91	0.86	0.96	*	0.81	0.70	0.94	*	0.65	0.61	0.69	*
PTSD	1.10	1.08	1.13	*	1.07	1.00	1.15	0.05	0.95	0.92	0.98	0.001
Blast-related conditions												
Postconcussive syndrome	0.90	0.85	0.95	*	0.81	0.69	0.94	0.005	0.64	0.60	0.68	*
Migraine	0.85	0.83	0.87	*	0.91	0.85	0.97	*	0.75	0.72	0.79	*

^*^p < 0.001.

### 3.2 Medical separation

Across all analyses, those working in high-risk occupations were significantly more likely to be medically separated from service than their lower risk peers (ORs ranged between 1.05 to 1.19; see Table 4). Additionally, those diagnosed with the conditions of interest were significantly more likely to be medically separated from service than those without the conditions of interest (ORs ranged between 1.11–7.07). The six diagnoses of interest with the largest odds of medical separation were: severe TBI (OR = 7.07), penetrating TBI (OR = 5.26), gait and coordination problems (OR = 4.68), skin sensation disturbances (OR = 3.50), bipolar disorder (OR = 3.47), and communication problems (OR = 3.05).

**Table 4:** Results from Cox regression analyses showing associations of occupational risk for LLB, diagnoses, and their interaction on medical separations when adjusting for number of years in the study window, branch of service, and sex.

	Occupational LLB risk				Diagnosis				Interaction			
	OR	95% CI LL	95% CI UL	*p*	OR	95% CI LL	95% CI UL	*p*	OR	95% CI LL	95% CI UL	*p*
TBI												
Any TBI	1.13	1.12	1.14	*	1.69	1.66	1.72	*	1.19	1.17	1.21	*
Mild TBI	1.14	1.13	1.15	*	1.60	1.57	1.63	*	1.18	1.16	1.20	*
Moderate TBI	1.16	1.15	1.17	*	2.74	2.67	2.82	*	1.12	1.08	1.15	*
Severe TBI	1.19	1.18	1.20	*	7.07	6.38	7.85	*	0.89	0.80	0.99	0.04
Penetrating TBI	1.19	1.18	1.20	*	5.26	4.77	5.80	*	1.05	0.95	1.16	0.35
Conditions comorbid with concussion												
Altered mental status	1.18	1.17	1.19	*	2.57	2.49	2.65	*	1.07	1.03	1.10	*
Cognitive problems	1.14	1.14	1.15	*	2.00	1.96	2.04	*	1.17	1.14	1.19	*
Communication problems	1.18	1.17	1.19	*	3.05	2.89	3.21	*	1.15	1.09	1.21	*
Dizziness/vertigo	1.18	1.17	1.19	*	1.89	1.79	1.98	*	1.31	1.24	1.38	*
Gait and coordination problems	1.19	1.18	1.20	*	4.68	4.22	5.19	*	1.03	0.93	1.15	0.58
Headache	1.14	1.13	1.15	*	1.97	1.93	2.00	*	1.22	1.20	1.24	*
Hearing problems	1.16	1.15	1.17	*	1.52	1.49	1.54	*	1.06	1.04	1.08	*
Nonheadache pain	1.05	1.03	1.07	*	2.13	2.08	2.17	*	1.18	1.15	1.20	*
Skin sensation disturbances	1.19	1.18	1.20	*	3.50	3.19	3.85	*	1.20	1.09	1.32	*
Sleep disorders and symptoms	1.11	1.09	1.12	*	2.13	2.10	2.16	*	1.19	1.17	1.20	*
Syncope and collapse	1.19	1.18	1.20	*	2.33	2.16	2.52	*	1.11	1.02	1.20	0.01
Vision problems	1.18	1.17	1.19	*	1.11	1.08	1.15	*	1.15	1.11	1.19	*
Mental health conditions												
Any behavioral health condition	1.12	1.11	1.13	*	2.13	2.10	2.16	*	1.10	1.08	1.11	*
Adjustment disorders	1.15	1.14	1.17	*	1.97	1.94	2.00	*	1.11	1.10	1.13	*
Anxiety disorders	1.14	1.13	1.15	*	2.18	2.15	2.21	*	1.14	1.12	1.16	*
Acute stress disorders	1.19	1.18	1.20	*	2.97	2.61	3.39	*	1.38	1.20	1.59	*
Alcohol abuse/dependence	1.18	1.17	1.19	*	1.14	1.12	1.16	*	1.02	1.00	1.04	0.09
ADD/ADHD	1.18	1.17	1.19	*	1.41	1.37	1.46	*	1.10	1.07	1.13	*
Bipolar disorder	1.19	1.18	1.20	*	3.47	3.35	3.59	*	1.02	0.99	1.06	0.24
Delirium/dementia	1.15	1.15	1.16	*	2.69	2.62	2.77	*	1.20	1.16	1.23	*
Depression	1.17	1.16	1.18	*	2.68	2.64	2.73	*	1.12	1.10	1.14	*
Drug abuse/dependence	1.18	1.18	1.19	*	1.66	1.61	1.70	*	1.02	0.99	1.05	0.16
Personality disorders	1.19	1.18	1.20	*	2.64	2.54	2.74	*	1.09	1.05	1.14	*
PTSD	1.11	1.10	1.13	*	2.78	2.74	2.83	*	1.17	1.15	1.19	*
Blast-related conditions												
Postconcussive syndrome	1.16	1.15	1.16	*	2.61	2.54	2.69	*	1.22	1.18	1.26	*
Tinnitus	1.17	1.16	1.18	*	1.70	1.67	1.74	*	1.04	1.02	1.06	*
Fatigue	1.17	1.16	1.18	*	1.51	1.48	1.54	*	1.18	1.15	1.20	*
Migraine	1.16	1.15	1.17	*	1.97	1.94	2.01	*	1.25	1.23	1.28	*

^*^p < 0.001.

Significant interactions of occupational risk and diagnosis were observed for all diagnoses except severe TBI, penetrating TBI, gait and coordination problems, syncope and collapse, bipolar disorder, and drug abuse/dependence. This suggests that the association between diagnoses for these conditions and medical separation was not significantly greater for those working in high-risk occupations than lower-risk occupations.

However, significant interactions emerged for all of the other diagnoses of interest (see Table 5). When decomposed as a function of occupational risk, the data suggest that each diagnosis of interest (with the exception of tinnitus, which had overlapping confidence intervals) had a stronger association with medical separation among those working in high-risk occupations than their lower risk counterparts. For example, the association between mild TBI and medical separation was significantly greater for those in high-risk occupations (OR = 1.91) than those in moderate (1.31) and low-risk (OR = 1.38) occupations. Additionally, those with (vs. without) postconcussive syndrome were 3.21 times more likely to be medically separated when they worked in a high-risk occupation compared to 2.19 or 2.06 times more likely when they worked in a low- or moderate-risk occupation, respectively. As discussed with the findings for administrative separation previously, we note that planned comparisons were not always statistically significantly different at each level.

**Table 5:** Decomposition of significant interactions of occupational risk of LLB and diagnoses of interest on medical separations when adjusting for number of years in the study window, branch of service, and sex.

Effect of diagnosis among	Low LLB risk				Moderate LLB risk				High LLB risk			
	OR	95% CI LL	95% CI UL	*p*	OR	95% CI LL	95% CI UL	*p*	OR	95% CI LL	95% CI UL	*p*
TBI												
Any TBI	1.44	1.40	1.47	*	1.40	1.32	1.49	*	2.04	1.99	2.10	*
Mild TBI	1.38	1.34	1.41	*	1.31	1.23	1.40	*	1.91	1.86	1.97	*
Moderate TBI	2.48	2.37	2.58	*	2.55	2.30	2.82	*	3.01	2.90	3.13	*
Conditions comorbid with concussion												
Altered mental status	2.43	2.34	2.52	*	2.37	2.14	2.63	*	2.73	2.59	2.87	*
Cognitive problems	1.73	1.68	1.79	*	1.63	1.51	1.76	*	2.37	2.29	2.44	*
Communication problems	2.69	2.49	2.91	*	2.86	2.36	3.47	*	3.38	3.13	3.66	*
Dizziness/vertigo	1.49	1.41	1.58	*	1.36	1.16	1.59	*	2.57	2.35	2.81	*
Headache	1.64	1.61	1.67	*	1.56	1.48	1.64	*	2.47	2.40	2.54	*
Hearing problems	1.45	1.41	1.49	*	1.44	1.35	1.54	*	1.57	1.53	1.62	*
Nonheadache pain	1.85	1.81	1.89	*	1.55	1.46	1.64	*	2.72	2.62	2.81	*
Skin sensation disturbances	2.93	2.60	3.30	*	3.61	2.66	4.91	*	4.12	3.51	4.83	*
Sleep disorders and symptoms	1.81	1.78	1.84	*	1.65	1.58	1.72	*	2.69	2.62	2.76	*
Vision problems	0.99	0.96	1.02	0.53	0.91	0.83	1.00	0.05	1.31	1.24	1.39	*
Mental health conditions												
Any behavioral health condition	1.96	1.93	1.99	*	1.73	1.66	1.80	*	2.46	2.39	2.52	*
Adjustment disorders	1.78	1.74	1.81	*	1.60	1.52	1.68	*	2.31	2.25	2.37	*
Anxiety disorders	1.93	1.90	1.97	*	1.78	1.70	1.86	*	2.57	2.50	2.64	*
Acute stress disorders	2.23	1.88	2.64	*	1.91	1.23	2.97	0.004	4.49	3.62	5.57	*
ADD/ADHD	1.29	1.25	1.34	*	1.27	1.15	1.40	*	1.59	1.51	1.67	*
Delirium/dementia	2.30	2.20	2.40	*	2.16	1.95	2.39	*	3.24	3.12	3.37	*
Depression	2.43	2.38	2.48	*	2.15	2.03	2.27	*	3.10	3.01	3.20	*
Personality disorders	2.45	2.35	2.54	*	2.19	1.96	2.45	*	3.02	2.82	3.25	*
PTSD	2.39	2.34	2.45	*	2.33	2.20	2.46	*	3.30	3.21	3.39	*
Blast-related conditions												
Postconcussive syndrome	2.19	2.10	2.29	*	2.06	1.85	2.29	*	3.21	3.09	3.34	*
Tinnitus	1.65	1.60	1.70	*	1.63	1.51	1.76	*	1.74	1.69	1.80	*
Fatigue	1.31	1.29	1.34	*	1.24	1.17	1.32	*	1.82	1.75	1.89	*
Migraine	1.61	1.58	1.65	*	1.57	1.48	1.66	*	2.54	2.46	2.63	*

^*^p < 0.001.

## 4 Discussion

Although there is a large body of research on the long-term outcomes associated with TBI, relatively limited research has directly examined whether TBI and associated conditions may affect one’s ability to maintain their military employment (3, 7–11, 43). Drawing on the increased recognition of the prevalence and adverse outcomes associated with TBI among military populations (15, 19, 21), the present research sought to examine whether military personnel diagnosed with TBI and associated conditions were at greater risk of military involuntary job loss for medical or administrative reasons. Furthermore, because LLB exposure has been identified as one potential risk factor for TBI among military personnel (35), the present research also examined whether those with greater occupational risk of LLB exposure were more likely to show these adverse career outcomes than their lower risk counterparts. To do this, population-level data from 2005–2015 were used to examine associations between TBI, conditions commonly comorbid with concussion, mental health conditions, blast-associated conditions, and occupational risk of LLB on likelihood of involuntary job loss for medical and administrative reasons.

### 4.1 Findings regarding medical diagnoses

This research shows that having been diagnosed with any TBI, concussion, and moderate TBI were significantly associated with greater risk of being medically separated from service. They were also significantly less likely to be administratively separated from service, presumably because medical reasons for separation took precedence over administrative reasons, as the two determinations are mutually exclusive. Severe and penetrating TBIs were also significantly associated with being medically, but not administratively, separated from service. Conditions that are often comorbid with concussion in military populations were associated with greater risk of medical separation and were generally associated with less risk of being administratively separated, which is consistent with changes in military policy over time that aim to ensure that service members are eligible for post-service benefits when appropriate. However, diagnoses of altered mental status and syncope and collapse were associated with greater risk of administrative separation. Although surprising, it is possible that positive drug screens were obtained at the time SMs received care for altered mental status or syncope and collapse, which would likely result in administrative separation from service over medical separation from service.

Additionally, all of the behavioral health conditions examined were associated with increased risk of being both administratively and medically separated from service, which appeared consistent with military policy. For example, those with diagnoses of drug abuse/dependence were more than 5 times more likely to be administratively separated from service and only 1.66 times more likely to be medically separated from service. Conversely, whereas those with (vs. without) PTSD were 2.78 times more likely to be medically separated from service; PTSD was only associated with a 1.03 times greater risk of being administratively separated from service, which is an effect size too small to be practically meaningful. Finally, the four blast-associated conditions examined were each associated with greater risk of medical separation and reduced risk of administrative separation. For example, those diagnosed with postconcussive syndrome or migraines were 2.61 or 1.97 times more likely to be medically separated from service, respectively, than those without these diagnoses on record.

The present research is the first to demonstrate involuntary military job loss outcomes associated with TBI, mental health conditions, conditions associated with blast exposure using population-level data that includes both inpatient and outpatient medical records. Whereas previous research demonstrated adverse career outcomes among those hospitalized with TBI, the present research suggests that such outcomes are not limited to injuries severe enough to require hospitalization but may also occur with concussions diagnosed in outpatient settings. Although these specific analyses were conducted on a military sample, these findings have important implications for both military and civilian workplace environments. If TBI inhibits an employee’s ability to perform adequately in the workplace, the associated costs may be much larger than previously estimated when considered in the context of additional costs to employers (e.g., to recruit and train new staff), to the employee (e.g., lost earning potential and insurance coverage), and to society at large (e.g., long-term taxpayer funded healthcare) (43).

### 4.2 Findings on LLB

In addition to the findings described above, the present research examined whether those who work in occupations marked by increased risk of repetitive LLB exposure are significantly more likely to be medically or administratively separated from service. Generally speaking, when adjusting for the diagnoses of interest, occupational risk was associated with increased risk of medical and administrative separation from service, though the effect sizes were relatively small in nature. However, the association between diagnoses of 14 specific conditions (e.g., concussion, postconcussive syndrome, cognitive problems) and administrative separation was significantly lower among those working in high-risk occupations compared to their lower risk counterparts. However, service members in high-risk occupations were significantly more likely to be medically separated from service when diagnosed with 26 of the 33 conditions of interest. Taken together, these findings suggest that there may be an association between occupational risk of LLB and administrative and medical separations from service. It is particularly noteworthy that service members diagnosed with concussion, conditions associated with concussion, mental health conditions, and blast-associated conditions are significantly more likely to be medically separated from service when they work in occupations categorized as high (vs. lower) risk of repetitive LLB exposure.

Previous research suggests that service members with repetitive exposure to LLB are more likely to report a variety of subclinical symptoms including headaches, memory lapses and decrements in working memory, difficulty concentrating, and more (44–46). Although a substantial body of research is currently underway to assess performance outcomes (47), the present findings provide preliminary evidence that such performance outcomes could extend as far as an inability to perform one’s occupational duties and ultimately subsequent involuntary military job loss when the service member has sustained a TBI or associated diagnosis. These findings also underscore the importance of understanding the full range of outcomes associated with LLB exposure and subsequent medical and non-medical care required. For example, it is possible that additional resources may be required to care for Veterans with (vs. without) chronic LLB exposure, particularly in light of potential neurodegenerative effects as they age (48, 49). Because these findings suggest that chronic exposure to LLB may be associated with involuntary military job loss, it is also plausible that Veteran homelessness may be an additional outcome worthy of investigation (50). However, we suggest that additional research would be necessary in light of the relatively small effect sizes identified herein.

## 5 Limitations

These findings should be considered in light of several important limitations. The present research relied on MOS as a proxy for LLB exposure; precise measures of blast exposure (including both high-level blast [HLB] from incoming munitions and LLB from outgoing munitions (33)) are not available at the population level and were unable to be incorporated into the present analyses. However, MOS has been used successfully as a proxy for LLB in previous work and the corresponding limitations have been discussed at length elsewhere (34, 35, 40, 51). Additionally, the present research was only able to examine whether someone received a clinical diagnosis within the medical record; more nuanced data regarding the nature of their clinical presentation (e.g., specific symptoms, mechanism of injury) were unavailable. We were thus unable to directly compare whether TBIs were associated with different outcomes based on whether they were HLB- induced or impact-induced, which has been associated with differences in symptomology in the past (34, 37, 52). We were also unable to directly examine whether these diagnoses were identified as the primary reason for inability to meet one’s occupational requirements for medical reasons; it is possible that other diagnoses (e.g., amputation) than those examined herein were the cause of a service member’s medical disability. It was also not possible to ascertain whether there are differences in eligibility requirements across occupations at high vs. low risk of LLB exposure which may influence determinations about one’s medical ability to continue serving following an injury. Additionally, the data utilized in the present study was limited to medical records during military service, and therefore lacked the ability to assess lifetime exposures to TBI and mental health conditions before service. Furthermore, the present findings are specific to military job loss in general (which may potentially convey additional personal costs, such as lost wages, health insurance coverage, and housing); the present research was not able to investigate whether the medical conditions of interest were associated with adverse employment outcomes post-service.

## 6 Conclusion

Taken together, the present research suggests that TBI and associated medical conditions, as well as occupational risk of LLB, are associated with long-term adverse career outcomes including involuntary military job loss due to medical inability to perform one’s job duties or non-medical reasons. This research adds to a growing body of evidence regarding the health and performance related consequences of both TBI and working in an occupation at greater risk of sustaining exposure to LLB (44–46). These findings underscore the need to continue working toward mitigation, identification, and treatment of blast-related exposures among military populations. For example, these findings reinforce the need for continued monitoring and surveillance of blast exposure (both HLB and LLB) and emphasize the need to reduce or mitigate LLB exposure during operational and training environments when permitted by the mission. Additionally, these findings also provide further evidence of the potential burden of TBI on long-term employment, which may be applicable to civilians working in a non-military environment as well.

Developing a thorough understanding of the long-term consequences associated with TBI and LLB exposure (e.g., dementia, chronic traumatic encephalopathy) will be even more important particularly in light of relatively recent evidence that those with concussion experience an evolution of symptoms up to five years later (53). Additional monitoring and follow up care by medical providers may be warranted. Additionally, enhanced training on the nature of LLB exposure and potential ramifications for health and well-being for frontline medical providers may be useful so that providers can make informed decisions with regard to progressive return to activity following concussion or more severe neurological injury. Furthermore, because conditions associated with medical and administrative separations are influential in determining service-related disability ratings and eligibility for post-service benefits, these findings raise the question of whether those with the diagnoses examined herein (e.g., concussion) and/or those working in high-risk occupations are also more likely to be eligible for and/or seek care from the VHA and underscores the potential growing burden of LLB exposure on the VHA system.

## Data availability statement

The data analyzed in this study is subject to the following licenses/restrictions: data were obtained from the Career History Archival Medical and Personnel System which is maintained by the Naval Health Research Center. Due to agreements covering data sharing, CHAMPS data cannot be shared outside of NHRC researchers. Requests to access these datasets should be directed to JNB, jennifer.n.belding.civ@health.mil.

## Ethics statement

The studies involving humans were approved by Naval Health Research Center Institutional Review Board. The studies were conducted in accordance with the local legislation and institutional requirements. The ethics committee/institutional review board waived the requirement of written informed consent for participation from the participants or the participants' legal guardians/next of kin because it was not possible to contact all eligible participants for this population-level study.

## Author contributions

JNB: Conceptualization, Data curation, Formal analysis, Funding acquisition, Investigation, Methodology, Project administration, Resources, Supervision, Validation, Visualization, Writing – original draft, Writing – review & editing. JB: Data curation, Investigation, Methodology, Software, Visualization, Writing – review & editing. RE: Conceptualization, Investigation, Methodology, Project administration, Resources, Supervision, Writing – original draft, Writing – review & editing.

## Author disclaimer

I am a military service member or employee of the U.S. Government. This work was prepared as part of my official duties. Title 17, U.S.C. §105 provides that copyright protection under this title is not available for any work of the U.S. Government. Title 17, U.S.C. §101 defines a U.S. Government work as work prepared by a military service member or employee of the U.S. Government as part of that person’s official duties. Lab Report #22-37 was supported by the U.S. Army Medical Research and Material Command under work unit no. N1518. The views expressed in this presentation are those of the authors and do not necessarily reflect the official policy or position of the Department of the Navy, Department of Defense, nor the U.S. Government. The study protocol was approved by the Naval Health Research Center Institutional Review Board in compliance with all applicable Federal regulations governing the protection of human subjects. Research data were derived from an approved Naval Health Research Center Institutional Review Board protocol, number NHRC.2016.0024.
